# Retinomorphic optoelectronic devices for intelligent machine vision

**DOI:** 10.1016/j.isci.2021.103729

**Published:** 2022-01-01

**Authors:** Weilin Chen, Zhang Zhang, Gang Liu

**Affiliations:** 1 National Key Laboratory of Science and Technology on Micro/Nano Fabrication, Shanghai Jiao Tong University, Shanghai 200240, China; 2 Department of Micro/Nano Electronics, School of Electronic Information and Electrical Engineering, Shanghai Jiao Tong University, Shanghai 200240, China; 3 School of Microelectronics, Hefei University of Technology, Hefei 230601, China

**Keywords:** Computer science, Artificial intelligence, Devices

## Abstract

Biological visual system can efficiently handle optical information within the retina and visual cortex of the brain, which suggests an alternative approach for the upgrading of the current low-intelligence, large energy consumption, and complex circuitry of the artificial vision system for high-performance edge computing applications. In recent years, retinomorphic machine vision based on the integration of optoelectronic image sensors and processors has been regarded as a promising candidate to improve this phenomenon. This novel intelligent machine vision technology can perform information preprocessing near or even within the sensor in the front end, thereby reducing the transmission of redundant raw data and improving the efficiency of the back-end processor for high-level computing tasks. In this contribution, we try to present a comprehensive review on the recent progress achieved in this emergent field.

## Graphical abstract

**Figure:**
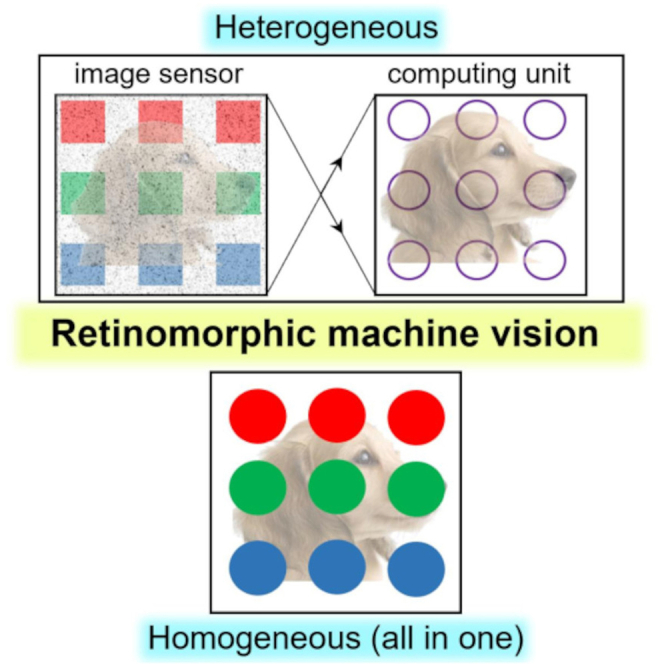

## Introduction

In the era of big data and the internet of things, the unprecedented huge amount of information and complex external environment put forward more stringent requirements for developing new-generation multi-functional artificial intelligence chips (Ham et al., 2021). Given that visual perception is one of the most important ways to obtain environmental information, the demand for visual information sensing, storage, and processing function devices with higher speed, greater efficiency, and lower power consumption is becoming ever more urgent. Although traditional machine vision technology has profoundly changed the lives of human beings in many fields, it has gradually become clumsy and inadequate, limited by the von Neumann bottleneck when dealing with complex tasks (Chai, 2020). Therefore, the development of more intelligent machine vision technology to satisfy the new requirements of the times has become one of the most important innovation directions in the field of artificial intelligence chips in the post-Moore era (Waldrop, 2016).

Human visual system is capable of visual information perception and multiple target recognition in complex environments, which inspires the development of biomimetic visual systems with new optoelectronic devices for high-performance machine vision technology (Abramoff et al., 2010). The main functions of the human visual system can be divided into two parts: image perception and preprocessing in the human eye and recognizing, memorizing in the visual center of the cerebral cortex. In recent years, several novel retinomorphic machine vision architectures have been developed and demonstrate strong vitality by simulating the working mechanism of the human visual system. According to different forms of functional divisions, heterogeneous and homogeneous integration architectures are the two main paradigms. Both architectures could perceive and preprocess the image information at the front end, thereby reducing redundant information and improving the overall recognition efficiency. Compared with traditional CMOS (complementary metal-oxide-semiconductor)-based machine vision systems, the novel retinomorphic optoelectronic devices exhibit obvious performance advantages. It has beendemonstrated that most energy consumption of traditional machine vision is spent on the redundant information transfer among the sensor, memory, and processor. Because the raw information can be preprocessed at the front end, the novel retinomorphic optoelectronic devices have inherent advantages in reducing energy consumption. The energy cost for writing information into memristors could also be reduced by more than 100 times. Yao and Wu et al. demonstrated that the energy consumption of electronic synapses is 1000 times smaller than the Intel Xeon Phi processor when dealing with similar face recognition tasks (Yao et al., 2017). The switching time (<10 ns), endurance (10^5^∼10^8^), and chip scaling potential (<10 ns) are also superior to those of the traditional counterparts (Ielmini and Wong, 2018; Milo et al., 2020). Therefore, retinomorphic optoelectronic devices may provide a new and effective approach for improving information processing efficiency and energy consumption problems in the era of big data.

Here, we present an overview of the recent advances in retinomorphic machine vision technology from principle to device. Firstly, the working mechanism of the human visual system and several differences with artificial retinomorphic devices are discussed. Then, two paradigms, *viz.* heterogeneous and homogeneous integration architectures, will be summarized and discussed in detail. Finally, a brief discussion on the current challenges and prospects of retinomorphic machine vision is provided.

## Biological basis of the retinomorphic machine vision system

**Figure 1:**
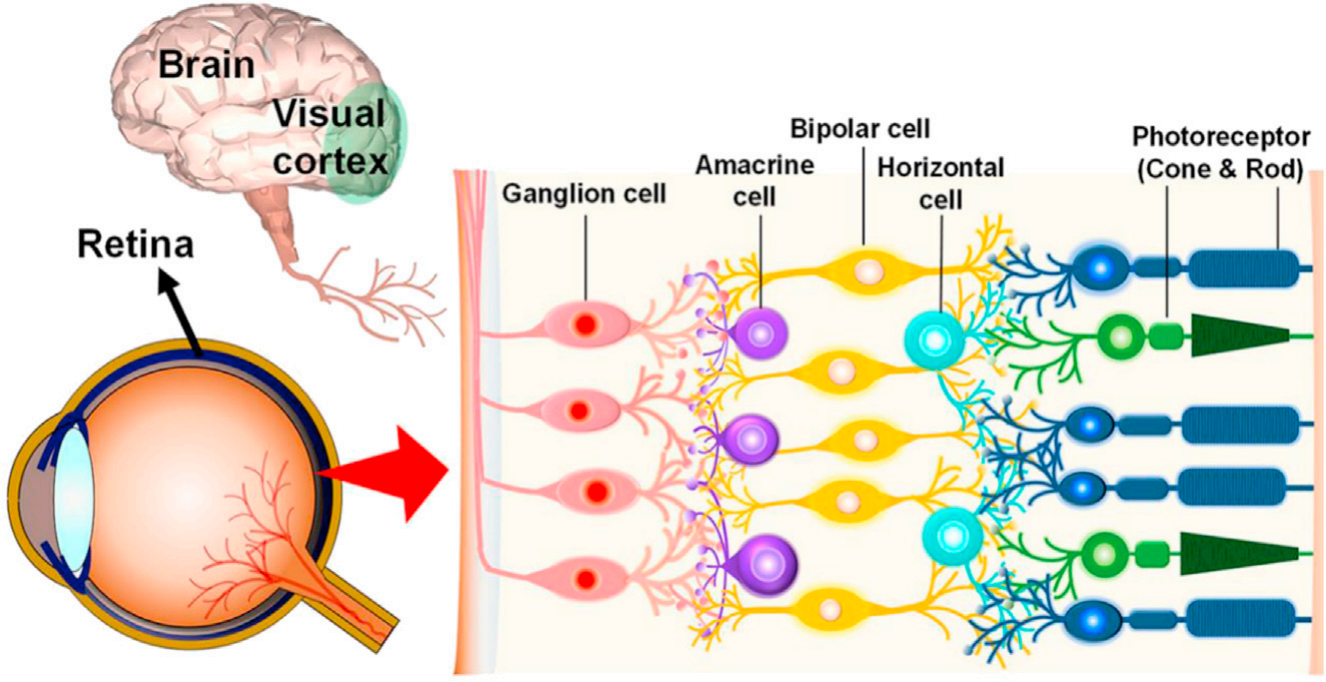
Schematic illustration of the biological visual perception system Reproduced with permission (Han et al., 2020). Copyright 2020, American Chemical Society.

Human visual system is capable of visual information perception and multiple target recognition in complex environments. It is demonstrated that human visual perception provides more than 80% of the information input in the process of human interaction with the surrounding environment and is one of the most important channels for humans to perceive external objects (Farrow et al., 2013). With the view of applied clinical anatomy, vision is a collaboration of the eyes and the brain (Shepherd et al., 2013). As shown in Figure 1, the human visual system mainly consists of the eyeballs, transmission nerve, and visual cortex of the brain. Light from the environment and external objects enters the crystalline lens through the pupil at the front of the eyeball, and finally reaches the retina after refraction (Han et al., 2020). In particular, the retina has a clear hierarchical structure for photoelectric information conversion, preprocessing, and transfer. There are 5 types of typical retinal cells, namely, ganglion cells, amacrine cells, bipolar cells, horizontal cells, and photoreceptor cells from outside to inside. During the photoelectric information conversion process, photoreceptor cells, namely, cone cells and rod cells, play a leading role in converting light signals into electrical signals (Hattar et al., 2003). There are more than 100 million rod cells with the same light-sensitive pigment within the human retina, which are dedicated to receiving dim light with high light sensitivity but can only identify the black or white outlines of objects, especially in night vision. As a comparison, approximately 7 million cone cells with different red, green, and blue light-sensitive pigments form the basis of human eye color vision to recognize the high-resolution environment under strong light (Abramoff et al., 2010). These two photoreceptor cells could efficiently perform the task of converting optical information of the environment into action potentials at the ends of axons and transmitting them further back, which is also the main work of the widely used optical sensors. After the photoelectric information conversion, the scattered electrical signals are integrated into bipolar cells, during which horizontal cells could regulate the physiological activities of photoreceptor cells within a reasonable range through negative feedback effect. In other words, the information preprocessing operation of the retina starts with bipolar cells and horizontal cells. The integrated electrical signals will be transferred to ganglion cells and regulated by amacrine cells, within which the redundant and unstructured visual data will be filtered out and the refined information is finally transmitted to the visual center of the brain through the optic nerve (Masland, 2012). This fine retinal cell hierarchical structure gives the human eye a variety of functions such as perception, signal classification and integration, preprocessing, etc.

It is noteworthy that the unique information transformation and preprocessing processes of the human retina in the front end can significantly reduce the burden on the visual cortex of the brain and ultimately accelerate the cognitive process of optical information (Gollisch and Meister, 2010; Guosong Hong et al., 2018). The discoveries of neuroscientists about the human visual system greatly inspire researchers to develop new generations of more intelligent machine vision chips for retinomorphic applications, aiming both to respond quickly to the complex external environment and combine multiple functions, *namely.* imaging, processing, and storage of visual information in the front end. Nevertheless, there are some differences between the human visual system and the reported retinomorphic optoelectronic devices in practical applications. The visual system of the human eye is structurally inverted, *i.e.,* the photoreceptor cells are at the bottom of the retina and the signal transfer and processing cells receive light stimulation firstly. This special cell hierarchical architecture is the product of evolution and can help photoreceptor cells get more nutrients from the retinal base. The practical architectures of artificial retinomorphic optoelectronic devices are contrary to the human eye to get the best photoresponse characteristics, especially in the heterogeneous integration scheme. The image sensor is generally exposed to light stimuli directly and the adjacent processor unit is arranged in a parallel manner or beneath it. Moreover, the biological cones and rod cells work in a complementary way in the human eye, which enables us to have accurate vision under different lighting conditions. There is generally only one kind of photosensitive medium within the specific retinomorphic optoelectronic device, and the dynamic photoconductivity can be regulated by the external field to realize the imaging of the complex environment. The information transfer and processing approach are also different. The functions of different cells within the retina are strictly divided, and the information transfer is mainly monodirectional from photoreceptor cells to ganglion cells. The input and output of the signals of artificial counterparts are determined according to the different working modes, and a single device can perform different functions at different working stages, *viz.,* functional diversity, especially in the homogeneous integration scheme. Understanding the similarities and differences between the human visual system and artificial retinomorphic optoelectronic devices will help develop more intelligent bionic chips.

## Advanced machine vision system with retinomorphic optoelectronic devices

**Figure 2:**
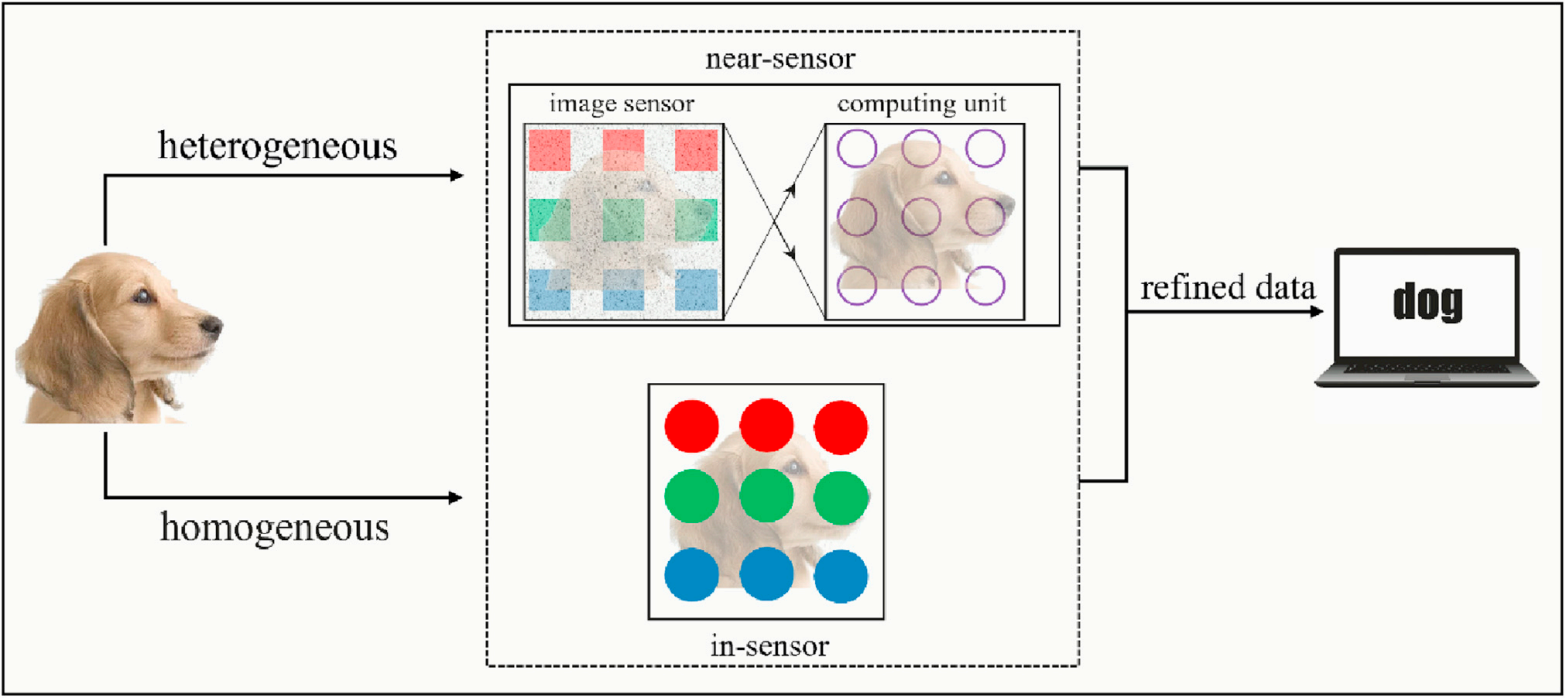
The illustration of heterogeneous integration and homogeneous integration machine vision system

**Figure 3:**
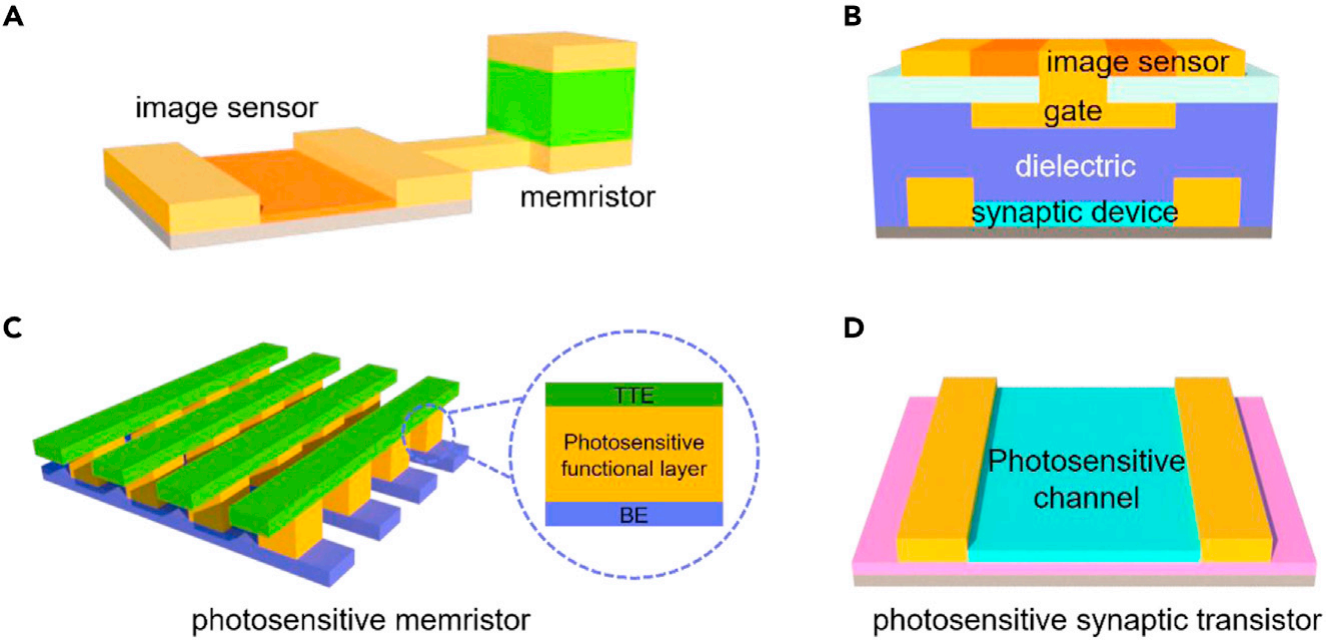
Schematics of possible configurations about retinomorphic devices (A and B) Planar and vertical heterogeneous integration architectures. (C and D) Photosensitive memristor-based and synaptic transistor-based homogeneous integration architectures.

Depending on whether the optoelectronic image sensors can perform *in-situ* preprocessing or not, the advanced machine vision system can be categorized into two families of heterogeneous integration and homogeneous integration, respectively (Figure 2). In the heterogeneous integration scheme, the image sensors can sense the visual information in a high fidelity manner, whereas the captured image is processed separately and accurately in the near-sensor-integrated neuromorphic computing units. In the homogeneous integration approach, the image sensors can both perform the adaptive visual sensing functions under varying illuminating conditions and execute the *in-situ* preprocessing tasks including denoising, edge enhancement, classification, recognition, etc (Chen et al., 2020; He et al., 2021). With the capability of in-sensor image preprocessing, the transmission of redundant raw data through the general von Neumann bottleneck between the front-end sensors and back-end processors will be greatly reduced, which in turn can significantly improve the efficiency for complex computing tasks (Zhou and Chai, 2020). Several typical retinomorphic device configurations are shown in Figure 3. For heterogeneous integration, planar and vertical series-mode frames of the image sensor and computing unit are widely used, which achieves the purpose of using light intensity to indirectly regulate the performance of the adjacent memristors or synaptic transistors (Figures 3A and 3B). It is worth mentioning that the image sensor must be directly exposed to light to obtain the best light response characteristics. Compared with the planar counterpart, vertical architecture processes the potential of smaller chip size, but higher requirements of the integrated process are needed. As for the homogeneous integration scheme, photosensitive memristors and synaptic transistors are the mainstream of the current research. From “in-memory computing” to “in-memory sensing and computing,” memristors have achieved vigorous development in the field of bionic electronics in recent years. The classic photosensitive memristor mainly consists of three parts: the top transparent electrode (TTE), the photosensitive resistive layer, and the bottom electrode (BE), which could be integrated into the crossbar array to perform complex tasks (Figure 3C). Compared with photosensitive memristors, the photosensitive channels of synaptic transistors are exposed to light directly, which means more ideal regulation effects (Figure 3D). Several reports of using ferroelectric materials as gate dielectrics or directly as photosensitive channels have attracted people's attention, which could enhance the retention characteristic of the devices after the stimulation removal (Wang et al., 2018a; Wang and Hu, 2017). More detailed introductions about different retinomorphic materials and devices are exhibited as follows.

### Retinomorphic optoelectronic devices and heterogeneous integration for near-sensor computing

Briefly, heterogeneous integration is the series-mode frame of the image sensor, information storage, and computing units to realize optical information perception and preprocessing at the near-stimulus end. Owing to the separation of different functions, the relatively mature image sensors can be directly compatible with the image processing unit through the ingenious circuit design, thereby realizing optical information preprocessing operations at the front end. In recent years, both image sensors and information processing units have developed rapidly.

#### Retinomorphic image sensing devices

**Figure 4:**
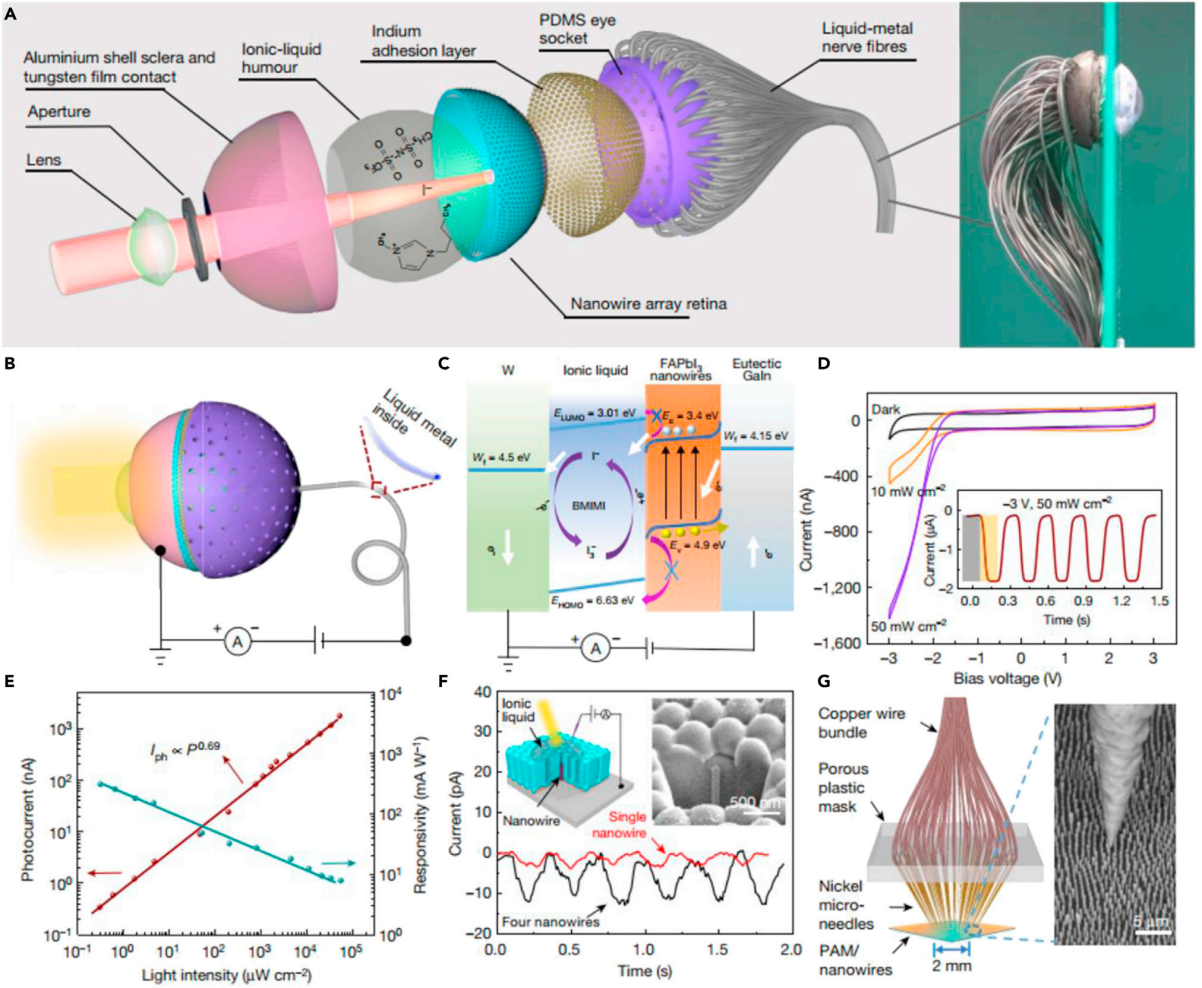
Structure and performances of the biomimetic eye (A) Schematic diagram and optical image of the structure of the artificial eyeball. (B) Schematic setup of individual pixel measurement. (C) Working mechanism and band structure of a single sensor unit. (D) Current-voltage curves under different illuminations. (E) Illumination-intensity-dependent photocurrent and responsivity of an individual pixel. (F) Device schematic and transient photoresponse of single-nanowire-based and four-nanowire-based individual pixels. (G) Schematic and scanning electron microscopy (SEM) image of the Ni microneedle contact to the nanowire array. Reproduced with permission (Gu et al., 2020). Copyright 2020, Springer Nature.

Converting light stimulus into transmittable electrical signals is the first step for the human visual system to process information, which is also the main function of various existing image sensors. In principle, millions of cone cells and rod cells work together to make the human eye have many attributes, such as high sensitivity, high-resolution, low-aberration, wide color gamut and field of view, self-adaptive ability, etc. By imitating the structure-function relationship of the human eye, scientists have done a lot of research from the perspective of materials and device structures, within which many works can match or even exceed the abovementioned functions of the human eye (Chow and Someya, 2020; Lan et al., 2020; Wang et al., 2020c). However, most researchers are either stuck on the illustration of single device functions, or lack a complete hardware supporting system to demonstrate the potential of practical applications. It is urgently needed to prepare an artificial retinomorphic sensor system comparable to the human eye through systematic collaborative innovation. Excitingly, an elaborate humanoid retinomorphic bionic eye based on the perovskite nanowire was proposed by Fan, which is a significant breakthrough in the field of the bionic eye in recent decades (Gu et al., 2020). As shown in Figure 4A, the prepared artificial eye completely mimics the primary structure of the human eye. The front optical lens, ionic liquid, perovskite nanowire array, and rear liquid metal fibers correspond to the crystalline lens, vitreous, rod cells, and nerve fibers of the human eye, respectively. The hemispherical aluminum oxide substrate can not only simplify the preparation process of the perovskite array, increase the density of the photoreceptor, but also give the artificial eye a wider field of view compared with the traditional planner devices. It is demonstrated that the density of the prepared perovskite array is as high as 4.6×10^8^ cm^−2^, which is more than four times the density of the rod cells of the human retina. Higher sensor density means higher image resolution and the rear design of liquid metal fibers also avoids visual blind spots. Figure 4B exhibits the overall shape and measurement setup of the artificial eyeball. As shown in Figure 4C, the separation of photogenerated carriers within the perovskite sensor and the simultaneous redox reaction at the electrode interface produce ordered and directional moving electrons and ions, respectively, which together constitute the detectable photocurrent. As shown in Figures 4D and 4E, the effective light intensity response range covers 0.3 μW cm^−2^ to 50 mW cm^−2^ and the maximum responsivity reaches 303.2 mA W^−1^, which indicates excellent light response characteristics compared with the reported counterparts. Although the density of perovskite photoreceptors is extremely high, the actual pixel size is limited to the order of millimeters by the minimum size of the signal transmission unit, *viz.,* liquid metal fiber. To further prove the application potential of the prepared artificial visual system in ultra-high pixel sensing, the photoelectric properties of single or several perovskite nanowires were investigated through the advanced micro-nano processing technologies and magnetic field-assisted positioning strategy, which shows distinguishably stable photocurrents (Figures 4F and 4G). Moreover, the final image sensing effect of the complete optical system, including artificial eye, circuit board, and signal processing terminal, was also successfully demonstrated. Although there are still several problems to be solved and optimized before actual application, such as how to deduce overall process costs, increase pixel density, improve the stability and service life of the perovskite-based sensor, optimize the concentration of ionic liquids, etc., this research is of great significance in inspiring scientists to develop more intelligent retinomorphic sensors and other bionic electronic devices.

#### Neuromorphic computing devices

Inspired by the development of brain neuroscience, neuromorphic computing is a brand-new computing model based on the structure of brain nerve circuits and the principle of neural impulse calculation, which is considered to be an effective way to solve the separation problem of storage and calculation based on von Neumann architecture (Boybat et al., 2018; Mead, 1990; von Neumann, 1993). In principle, the physiological activities of synapses and neurons are stimulated by the primary electronics and these elements work in a brain-like mode to decouple the above dilemma. Memristors, namely nonlinear memory devices with programmable resistance states, have become an important component of the construction of artificial neurons and synapses and indicated extensive parallelism and high-efficiency edge computing capabilities (Wang et al., 2017; Yao et al., 2020; Zidan et al., 2018). Moreover, when the nano-micro-scale memristors are integrated into the crossbar array, multiply-accumulate calculations could be performed simultaneously, *viz.,* executing multiplication and adding operations on each node and column based on Ohm's law and Kirchhoff's law, respectively (Burr et al., 2017; Hu et al., 2018; Zhang et al., 2020). Benefitting from the potential of “processing within memory,” memristors show a great significance for improving the information processing capacity at the front end of the hardware, especially in the era of information explosion (Wang et al., 2018b).

**Figure 5:**
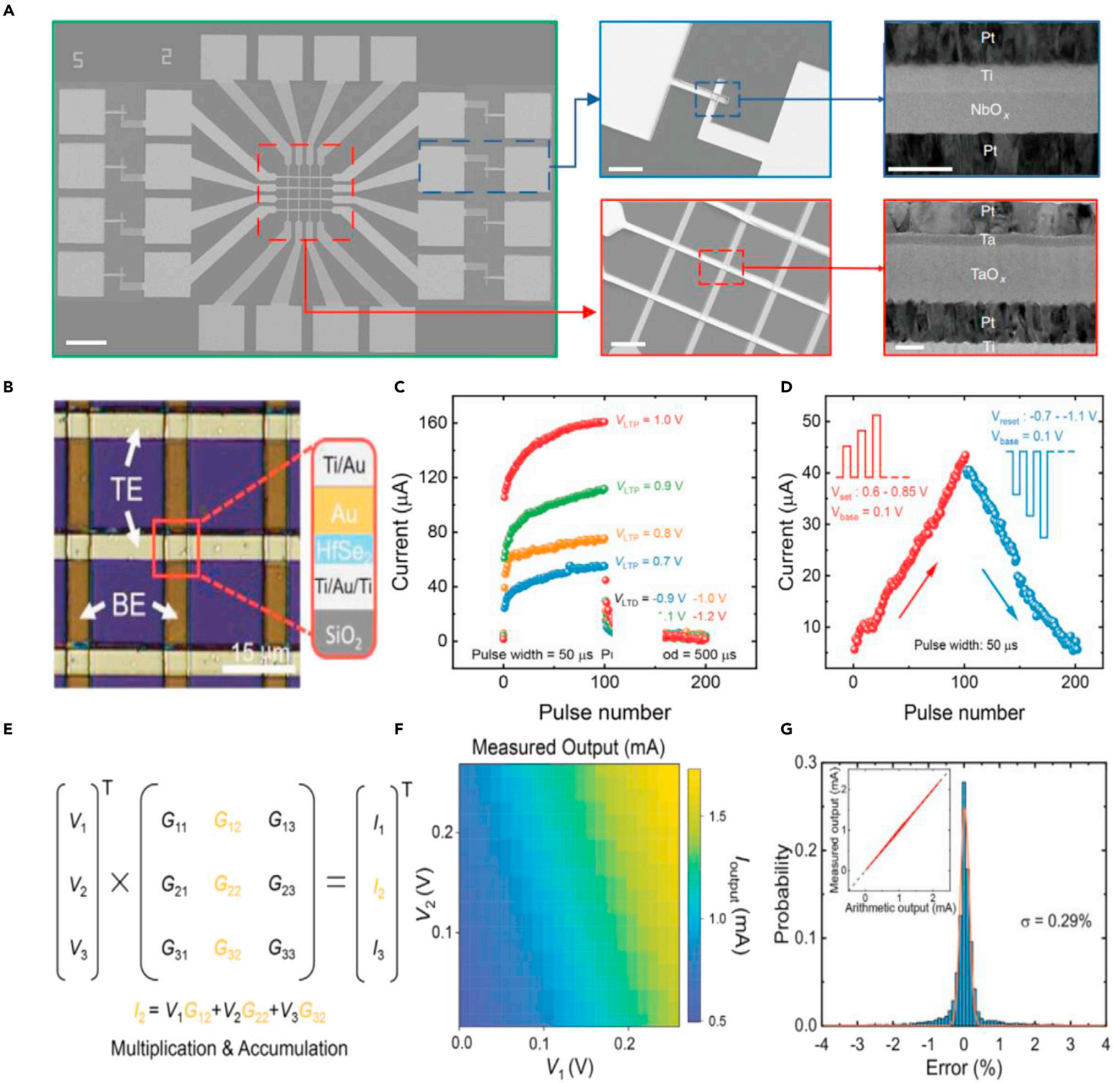
Memristors for neuromorphic computing (A) SEM images of the monolithically integrated memristive neural network. Reproduced with permission (Duan et al., 2020). Copyright 2020, Springer Nature. (B) Optical microscope image of the HfSe_2_-based crossbar array. (C) Long-term potentiation and depression under positive and negative pulse trains with identical pulse amplitude. (D) Long-term potentiation and depression under pulse train with nonidentical pulse amplitude. (E) The mathematical expression of multiply-accumulate operation. (F) Measured output current mapping under low resistance state conditions. (G) The corresponding distribution of error between measured and arithmetic results. Reproduced with permission (Li et al., 2021). Copyright 2021, John Wiley &Sons, Inc.

Lu et al. have done several pioneering research in the field of using memristor arrays to process complex information. In 2017, a 32 × 32 WO_*x*_ memristor array was fabricated and further deduced the application prospects of sparse coding algorithms in the field of image processing (Sheridan et al., 2017). Moreover, a more full-featured memristor neuromorphic chip with a memristor crossbar core and several peripheral control modules was developed, which not only provides a hardware platform for executing a variety of neural network algorithms but proves the good compatibility of memristor with conventional CMOS devices (Cai et al., 2019). Apart from synaptic simulation, memristors have also made significant progress in the field of neuronal simulation. Yang et al. proposed a fully memristive neural network based on NbO_*x*_ artificial neuron and TaO_*x*_ artificial synaptic array, which demonstrates the ability in image recognition (Figure 5A). The NbO_*x*_ artificial neuron shows several novel neuron behaviors such as spatiotemporal integration and multiplicative gain modulation (Duan et al., 2020). It is noteworthy that this full-memristor system is of great significance in expanding the application prospects of memristors in the releasing and processing of neural signals and inspiring scientists to develop more intelligent bionic electronic systems.

Most recently, Ang et al. proposed a HfSe_2_ based crossbar array to explore the application prospects of large-area two-dimensional (2D) materials in the field of memristors (Li et al., 2021). As shown in Figures 5B a four-layer device structure was established through molecular beam epitaxy and an elaborate thin-film transfer technology. Owing to the migration effect of the active electrode under the voltage bias, the prepared memristors exhibit excellent resistive switching properties, such as relatively small switching voltage and excellent cycle durability more than 500 times. Moreover, two typical synaptic properties, *viz.,* long-term potentiation and depression with excellent symmetry and states retention were also simulated (Figures 5C and 5D), which benefits to improve the accuracy of image recognition. Furthermore, to demonstrate the application potential of HfSe_2_ based crossbar array in multiply-accumulate operations, several electrical testing and simulation were carried out. Different from traditional software-level simulation, this work truly realizes the verification of the multiply-accumulate function at the hardware level. As shown in Figures 5E–5G, the output current of the array increased with the input voltage and exhibited a very small deviation compared with the simulated result, which indicates the potential to perform complex calculations at the hardware level in an efficient and ultralow-power consumption manner (8-trillion s^−1^ W^−1^).

#### Heterogeneously integrated machine vision system

Heterogeneous integration architecture of advanced machine vision, *viz.,* combining the optical sensor unit with the information storage and processing unit, has gradually become an important branch to improve the efficiency of optical information decoding and processing (Mukhopadhyay et al., 2019; Zhong et al., 2018). Compared with traditional optical sensors, this integrated architecture could directly store and process electrical signals near the image sensors, which could reduce the movement of data and improve the efficiency of image processing (Jang et al., 2021). Generally, when the image sensors and the memories or synaptic devices are connected in series, the voltage levels of these two parts will be redistributed under light bias. When the partial voltage bias of memories or synaptic devices reaches the transition threshold, the electrical status of the adjacent sensors will be recorded or further processed. Because the circuit design of this integration architecture is very simple, retinomorphic machine vision systems based on heterogeneous integrations have achieved vigorous development.

**Figure 6:**
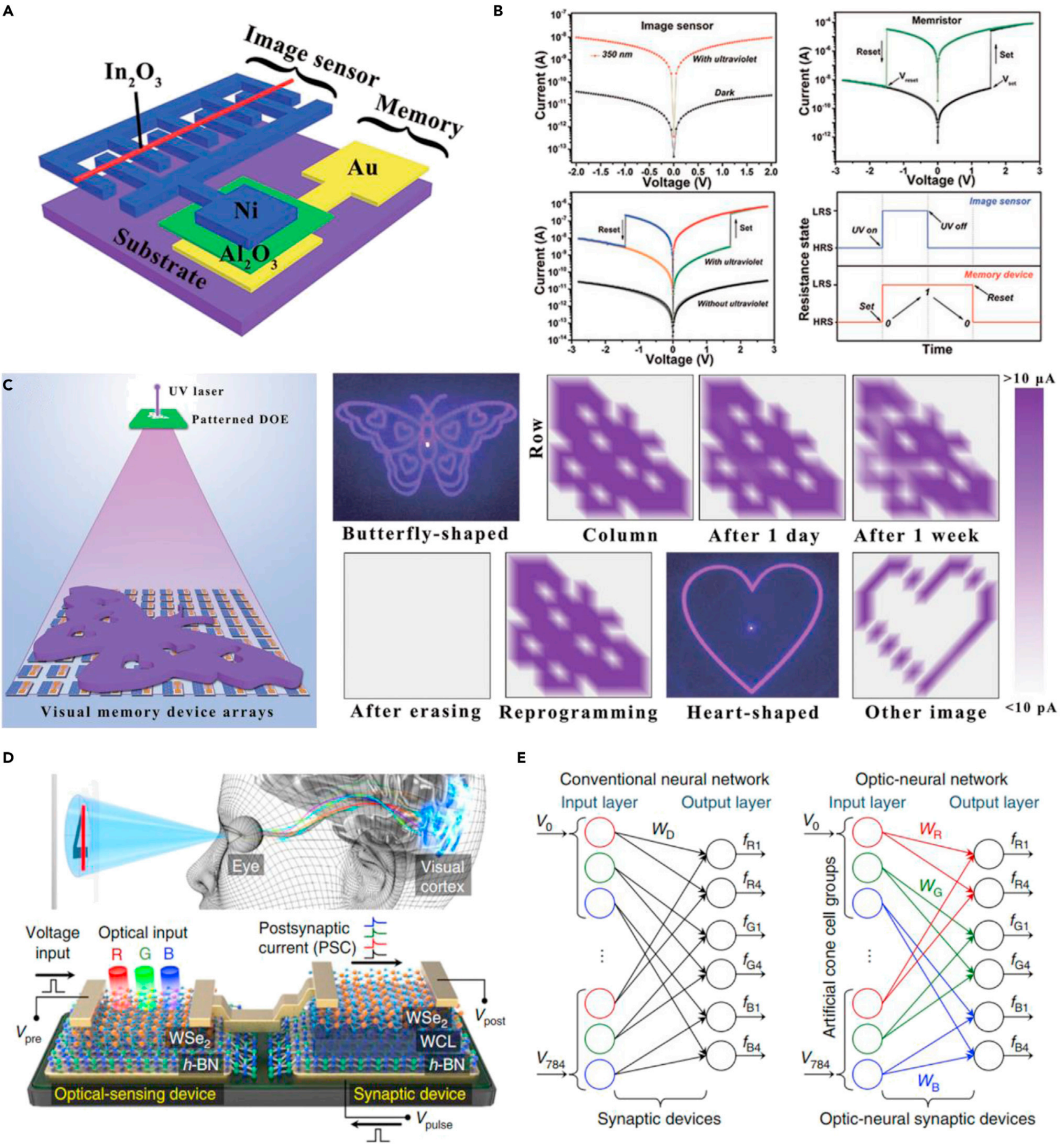
The heterogeneously integrated machine vision system (A) Schematic illustration of the prepared heterogeneous structure. (B) Current-voltage characteristics of the single unit and schematic illustration of resistance states between the image sensor and memory device. (C) Schematic diagram of the image capture and memory process. Reproduced with permission (Chen et al., 2018). Copyright 2018, John Wiley &Sons, Inc. (D) Schematic of the human optic nerve system and device structure diagram. (E) Optical-neural network based on optic-nerve synaptic devices compared with the conventional neural network. Reproduced with permission (Seo et al., 2018). Copyright 2018, Springer Nature.

Shen et al. have done pioneering work in solving the problem that optical signals cannot be stored in the front end (Chen et al., 2018). As shown in Figures 6A and 6B, because of excellent light response characteristics (*I*_*light*_*/I*_*dark*_ ratio up to 10^4^) and robust nonvolatile resistive-switching characteristics, In_2_O_3_ and Al_2_O_3_ were chosen as photosensitive material and resistive switching material, respectively. When these two units were connected in series and exposed to ultraviolet light, the partial voltage bias of the image sensor drops sharply, and the memory unit will obtain enough voltage to switch from the high resistance state to the low resistance state to realize the storage of optical signals. Moreover, the stored optical signals can be erased by electrical signals, thereby exhibiting multiple information sensing and storage capabilities. Furthermore, a 10 × 10 flexible visual memory array was prepared, which demonstrated excellent real-time ultraviolet (UV) distribution detection and long-term storage capabilities (Figure 6C). However, limited by the accuracy of printing technology, the lateral dimension of the microwire is greater than 5 μm. A more refined preparation technology is needed to develop higher density and resolution visual memory arrays, especially for high-quality detection of tiny objects. In addition, although this work solves the problem of perception and storage of optical information in the front end, the prepared visual memory system cannot perform information preprocessing functions compared with the human visual system. To solve this problem, Park et al. proposed a similar optical nerve synapse device using 2D material heterojunctions (Seo et al., 2018). Compared with Shen's work, a synaptic transistor was selected to replace the memory unit, which endows the new serial architecture the ability to perform neuromorphic computing by regulating synaptic plasticity in the front end. As shown in Figures 6D a typical WSe_2_/*h*-BN heterojunction was served as the optical-sensing unit and a specially processed WSe_2_/*h*-BN heterojunction with more trapping center was served as the synaptic device. Different wavelengths of light can make the optical-sensing device in different electrical conduction states, thus further regulating the synaptic dynamic properties of the synaptic transistor. Several typical synaptic properties were successfully demonstrated on the optical nerve synapse device. Moreover, the prepared WSe_2_/*h*-BN heterojunctions were applied to the optical nerve network to perform colored and color-mixed pattern recognition functions. As shown in Figure 6E, two neural networks were established to test the recognition function of mixed color numbers. Compared with the conventional neural network, the optical-sensing function was added to the new-style optical-neural network, thus not only reducing the complexity of the peripheral circuit but significantly improving the recognition rate of the target images.

Apart from the abovementioned series structure of electronic components with specific functions, the specially designed analog circuits could be also used as the image perception and processing modules. In 2020, Labram et al. proposed a simple photosensitive capacitor coupled with a resistor to simulate the human cognitive ability of dynamic scenes. The prepared analog circuit will output a voltage pulse only when the light intensity changes, which is consistent with the higher sensitivity of the human eye to sudden changes of light stimulation (Trujillo Herrera and Labram, 2020). This novel working mode can greatly reduce the redundant information volume from the source and has broad development prospects in some specific fields, such as intelligent security, abnormal alarm, etc. Moreover, compared with traditional circuit design, analog voltage signals were output directly instead of current signals, which could be accessed by subsequent neuromorphic devices without the need for transimpedance amplifiers. This work provides a new approach for scientists to simulate the cognitive function of the human eye from the perspective of analog circuit design.

It is worth mentioning that all these researches mentioned above use a combination of simple components to achieve the integrated functions of sensing, storing and computing in the front end, which is a critical step in simulating the main functions of the human visual system. However, the discrete architecture of the optical nerve synapse does not completely get rid of the shackles of the von Neumann architecture and indirect information transfer between the optical-sensing device and the synaptic transistor is still needed. Therefore, it is an urgent demand to develop large-scale integrated all-in-one devices to achieve a more efficient artificial vision system (Pedretti and Ielmini, 2021; Zidan et al., 2018).

### Retinomorphic optoelectronic devices and homogeneous integration for in-sensor computing

Homogeneous integration, that is, a single device is endowed with multiple functions such as image perception, storage, and preprocessing (Sebastian et al., 2020; Xia and Yang, 2019). By switching different working modes, it can perform different functions without outward transfer of information. Heterogeneous integration represents the development direction of intelligent machine vision technology, which fundamentally overcomes the constraints of von Neumann's bottleneck. In 2019, Chai et al. pioneered the research in demonstrating the abovementioned ‘all in one’ functions using MoO_*x*_-based memristors, which inspires researchers to develop more intelligent machine vision systems (Zhou et al., 2019). In addition to traditional metal oxide materials, several pioneering homogeneous machine vision technologies based on emerging materials have also achieved inspiring breakthroughs, within which 2D materials, perovskite materials, and organic materials attract the most attention.

#### 2D materials-based devices

2D atomic crystals and compound crystals have received great attention from the academic community since the discovery of graphene (Kang et al., 2020; Novoselov et al., 2016). It will trigger new research enthusiasm whenever a new type of layered material is found or synthesized, which is because of its strong light-matter interaction, intrinsic flexibility, as well as external tunability of device potential profile and performance by electrostatic doping, electrochemical regulation, and interface engineering, etc (Kang et al., 2020; Li et al., 2017; Mennel et al., 2020). In recent years, with the development of micro-nano processing technology and material synthesis methods, the wafer-level preparation of 2D materials has developed vigorously, which makes it possible to develop large-scale integrated retinomorphic optoelectronic devices (Claro et al., 2021; Hou et al., 2021; Tong et al., 2019).

The excellent photoelectric response characteristics and inherent persistent photoconductivity (PPC) effect or retention characteristic regulated by external fields endow 2D materials-based devices image perception and long-term memory functions (Kallatt et al., 2018; Shih et al., 2017). Because the conductance state of the 2D material-based transistor channel can be effectively adjusted by external stimuli, such as light, voltage bias, and magnetic field, the 2D materials-based photoelectric sensor array can perform an efficient image recognition function *in situ* when combined with an artificial neural network. Therefore, developing 2D materials-based optoelectronic devices to simulate the typical functions of the human visual system has gradually become a research hotspot in recent years (Hou et al., 2021; Tsai et al., 2021).

In 2020, a BN/WSe_2_ vertical heterojunction array was fabricated by Miao et al., which successfully simulated the optical information perception and preprocessing functions of cones and bipolar cells (Wang et al., 2020a). The photoconduction of the single heterojunction could be regulated by the gate voltage, thus reconfigurable image processing and recognition functions could be demonstrated at the hardware level. Most recently, Hao et al. proposed a ferroelectric heterojunction based on α-In_2_Se_3_/GaSe and the prepared device exhibited excellent photoelectric dual-regulation synaptic characteristics (Guo et al., 2021). Owing to the intrinsic ferroelectricity of α-In_2_Se_3_, logical operation and information storage functions were also demonstrated, which is a key breakthrough in using ferroelectric 2D material to improve the functions of artificial retinomorphic devices. However, this work only imitates the main functions of the human visual system from a single device perspective, and the photoelectric characteristics of the corresponding crossbar array as well as the demonstration of image recognition based on the artificial neural network are all simulated results. It is necessary to verify the comprehensive performance of the α-In_2_Se_3_/GaSe heterojunctions at the integrated hardware level before practical application. To explore the potential of 2D materials in the construction of neural photoelectric image sensor arrays, a reconfigurable 3 × 3 WSe_2_ photodiode array was fabricated by Muller et al. (Mennel et al., 2020). The prepared array shows excellent prospects to perform real-time image perception and processing functions at the nanosecond level. Two typical paradigms, *viz.,* classifier and autoencoder, were simulated by supervised and unsupervised learning, respectively. This work indicates the application potential of 2D materials-based photoelectric sensor arrays in short-latency and high-efficiency visual processing technology. However, an additional memory unit is needed to store the final processing results, which increases the complexity of the peripheral circuit. Appropriate external field regulation strategies or interface engineering may help to endow the device information storage capability. In addition, it is necessary to fabricate a larger array to deduce richer image processing functions.

**Figure 7:**
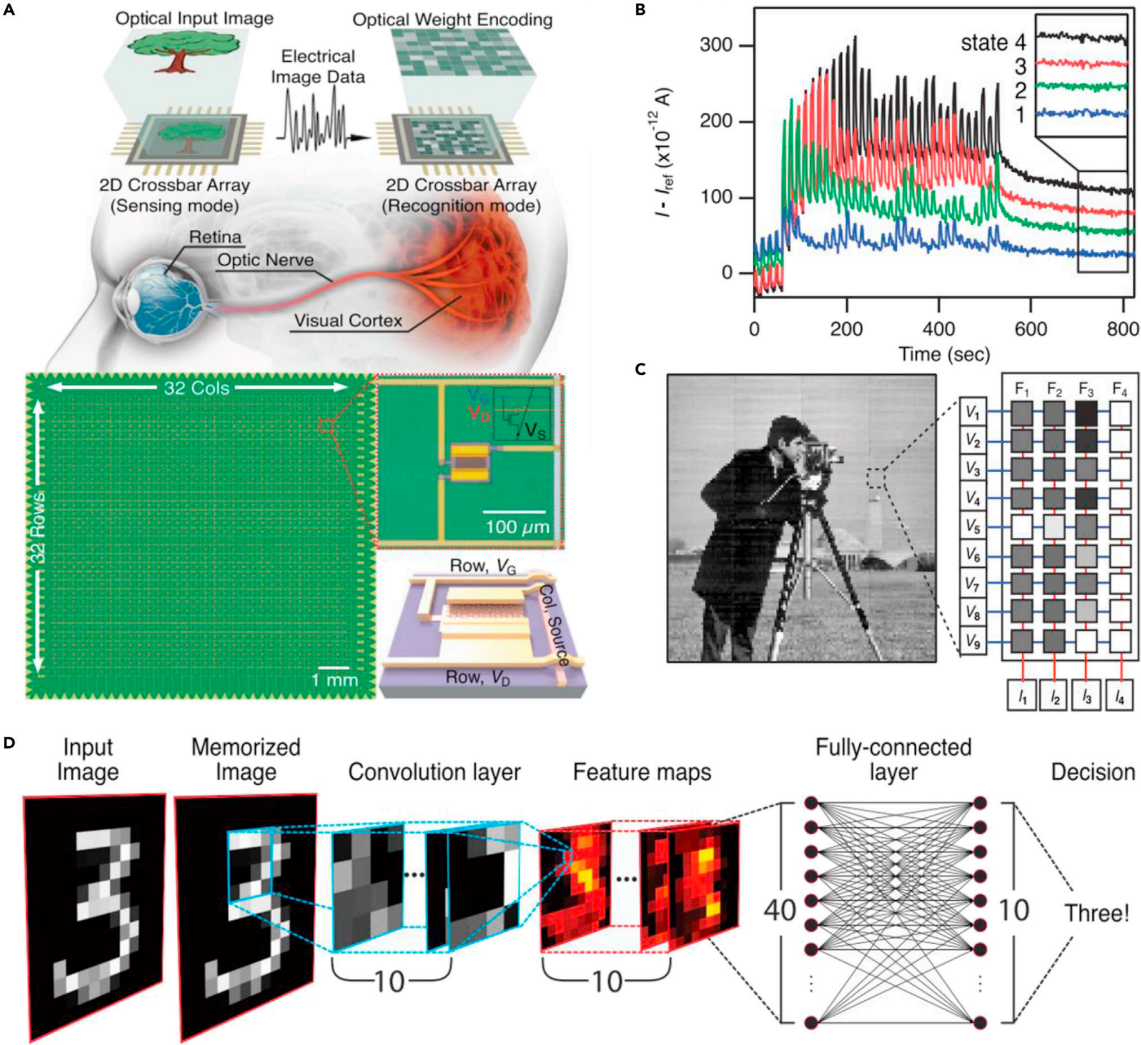
MoS_2_-based homogeneous integration machine vision system (A) Schematic illustrations of the human visual system and the prepared MoS_2_ crossbar array. (B) Iterative encoding of four pixels. (C) Schematic illustration of the image filtering with the 9 × 4 conductance matrix. (D) Image sensing and recognition process for an example of hand-written digit “3”. Reproduced with permission (Jang et al., 2020). Copyright 2020, John Wiley &Sons, Inc.

To further improve the integration density of phototransistors, explore the application potential of 2D materials in multifunctional machine vision technology, Ham and Park coreported a 32 × 32 MoS_2_-based phototransistor array with the functional complexity reaching an unprecedented level (Jang et al., 2020). As shown in Figure 7A, the prepared crossbar array simulated the dual functions of the human eye and brain by switching the two working modes of the crossbar array, *viz.,* sensing and recognition. Firstly, the MoS_2_-based phototransistor array was set to sensing mode to capture and store optical images of the environment because of the excellent photosensitivity of the MoS_2_ and PPC effect. It is worth mentioning that the conductance values of all transistors could be read within one second through exquisite external circuit design. Secondly, external voltage bias was applied to the gate to erase the stored conductance value of each transistor unit, *viz.,* initializing the entire array. Thirdly, a distinguishable conductance value was assigned to each transistor by optical encoding (Figure 7B), thereby a new conductivity matrix was built to perform image processing and recognition tasks. As shown in Figures 7C 4 different conductance matrices representing different processing factors, were established to filter the obtained image. Moreover, the prepared transistor array successfully simulated the high-level digital image recognition function by combining the convolutional neural network (CNN). As shown in Figure 7D, two initialization and re-encoding processes were performed to execute the matrix multiplication function after image capture. The first mode switch is to obtain the feature maps of the image and the second is to use the array as the fully-connected layer of the neural network to finally identify the digital information. In general, this work has taken a critical step in the field of high-density integration of 2D materials in the human-like image processing hardware field. There are still several technical issues worthy of further optimization. Firstly, compared with the image capture process, the complexity and time consumption of the optical encoding process (exceeding 10 min) are significantly increased, which may slow down the overall image processing speed. Moreover, multiple offline learning procedures in the software are needed to generate the required conductance values. However, this training process is difficult to expand to big data scenarios, thus limiting the range of image recognition on the hardware side. Faster encoding and smarter training methods can further improve the overall performance of the transistor array.

#### Perovskite materials-based devices

Perovskites have emerged as a rising star in various optoelectronic devices because of their unique crystal structure and rich physical properties, such as high carrier mobility and photoelectric conversion efficiency, tunable bandgap, intrinsic ambipolar transport, organic dispersibility, etc. (Kim et al., 2020; Samuel D. Stranks et al., 2013; Tian et al., 2020) Duan et al. reported a low-temperature solution growth process in 2015, which enables the large-scale preparation of perovskite-based optoelectronic devices (Wang et al., 2015). Till now, perovskite-based bionic machine vision devices have achieved vigorous development.

**Figure 8:**
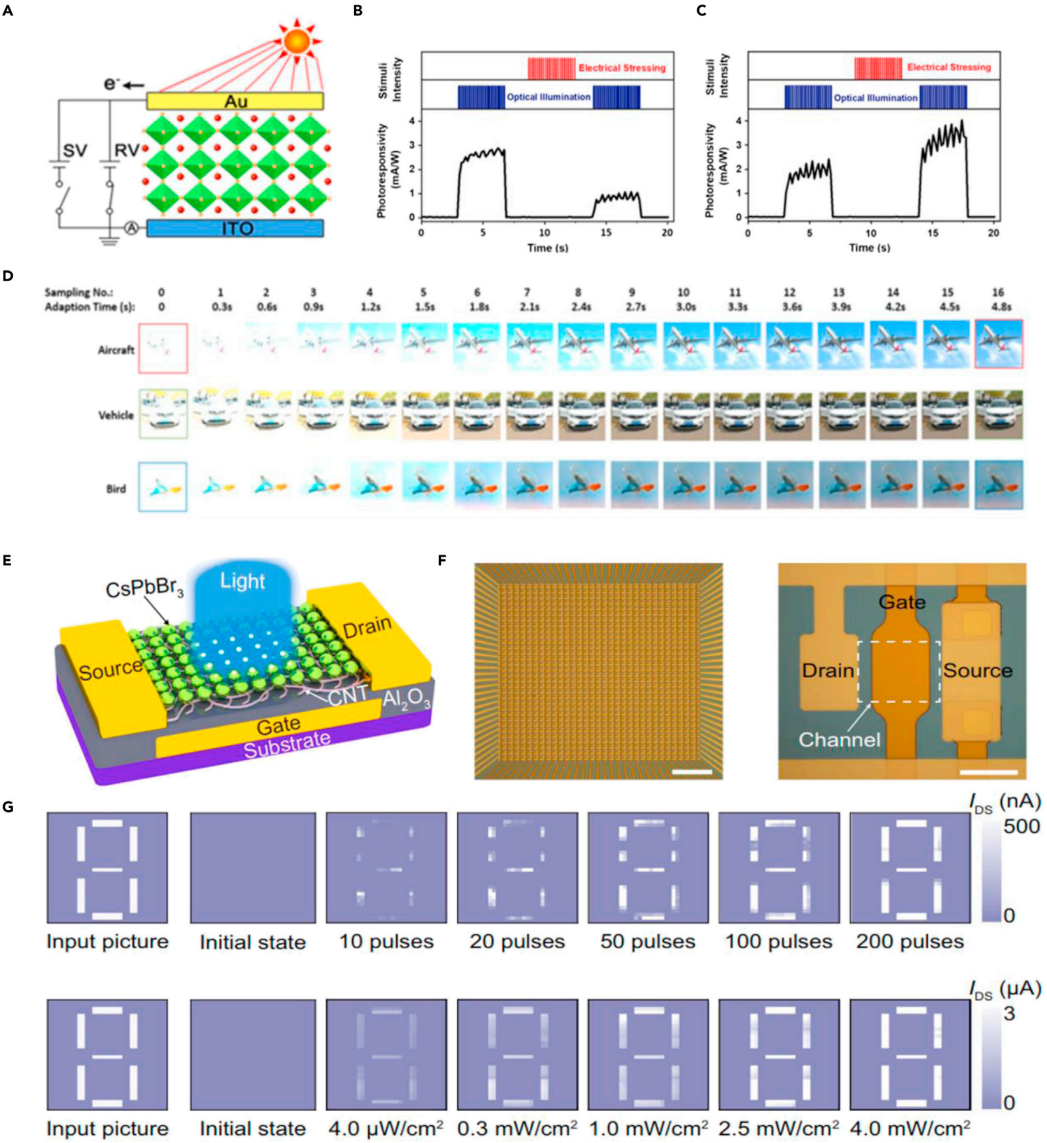
Perovskite-based homogeneous integration machine vision system (A) Schematic illustration of the prepared perovskite-based memristor. (B and C) Regulations of the device photoresponsivity by constant voltage stress. (D) Spontaneous adaptation of theoverexposed images with experimental dynamics of the spontaneous photoresponsivity relaxation. Reproduced with permission (Chen et al., 2020). Copyright 2020, John Wiley &Sons, Inc. (E) Schematic of the CsPbBr_3_/carbon nanotube-based phototransistor. (F) Optical micrograph of a 32 × 32 sensor array and the magnified image of an individual sensor unit. (G) Measured training weight results of a number 8 pattern along with increasing pulse number and light intensity. Reproduced with permission (Zhu et al., 2021). Copyright 2021, Springer Nature.

Owing to the fixed photosensitivity, traditional silicon-based photoelectric sensors lack high-precision imaging capabilities under extreme conditions, such as the excessively bright or dim environment (Fossum and Hondongwa, 2014). To improve this problem, Liu et al. proposed a self-adaptive retinomorphic system based on perovskite memristor (Figure 8A), which could perform “sensor-memory-processor” all in one function and image quality optimization purpose (Chen et al., 2020). As shown in Figures 8B and 8C, both light and electric bias could regulate the responsivity of the device because of external field-induced ion migration within the perovskite film. Moreover, a multilayer perceptron neural network (PNN) was established to perform instant computation tasks. As shown in Figure 8D, after filtrating the background noise by the perovskite-based memristor self-adaptively, the overexposed images of the aircraft, vehicle, and bird (with similar morphological characteristics) could be distinguished effectively with a maximum of 263% enhanced accuracy. However, the multiply-accumulate operation and neuromorphic computing functions based on the perovskite memristor array are the results of the simulation, which deserve real array-level verification before practical application. To explore the application prospects of perovskite in large-scale integrated photoelectric bionic devices, Sun et al. proposed a 32 × 32 flexible optoelectronic transistor array based on perovskite quantum dots and carbon nanotubes (Figures 8E and 8F), which combines the functions of the photodetector and synaptic electronic with high responsivity and synaptic plasticity regulation ability (Zhu et al., 2021). As shown in Figure 8G, both the number and intensity of light pulses can gradually increase the conductivity of the array, which exhibits the human brain-like reinforcement learning function. This is the first demonstration of reinforcement learning by ultra-low light pulses through a highly integrated physical device array. However, apart from reinforcement learning, forgetting is also a very important function of the human brain. Using electrical pulses to weaken the captured signal gradually at the hardware level also deserves further research.

#### Organic materials-based devices

Compared with other material systems, organic materials provide scientists with an enormous design platform to create novel molecules to meet specific functional requirements. In recent years, organic materials-based optoelectronic devices, especially photodetectors and luminescent devices, have developed vigorously because of tunable optoelectronic properties, low temperature processability, flexible, and stretchable properties and so on (Chow and Someya, 2020; Sun et al., 2019; Zhang et al., 2018). However, in the field of intelligent machine vision, most organic optoelectronic devices are dedicated to optoelectronic performance optimization at the single device level, and the exploration of the integrated machine vision system is insufficient. Although some progress has been made in neuromorphic computing based on organic electronics (van de Burgt et al., 2018), collaborative innovations from image perception, storage to computing functions to develop new types of human-like vision chips based on organic materials are facing a more urgent demand.

**Figure 9:**
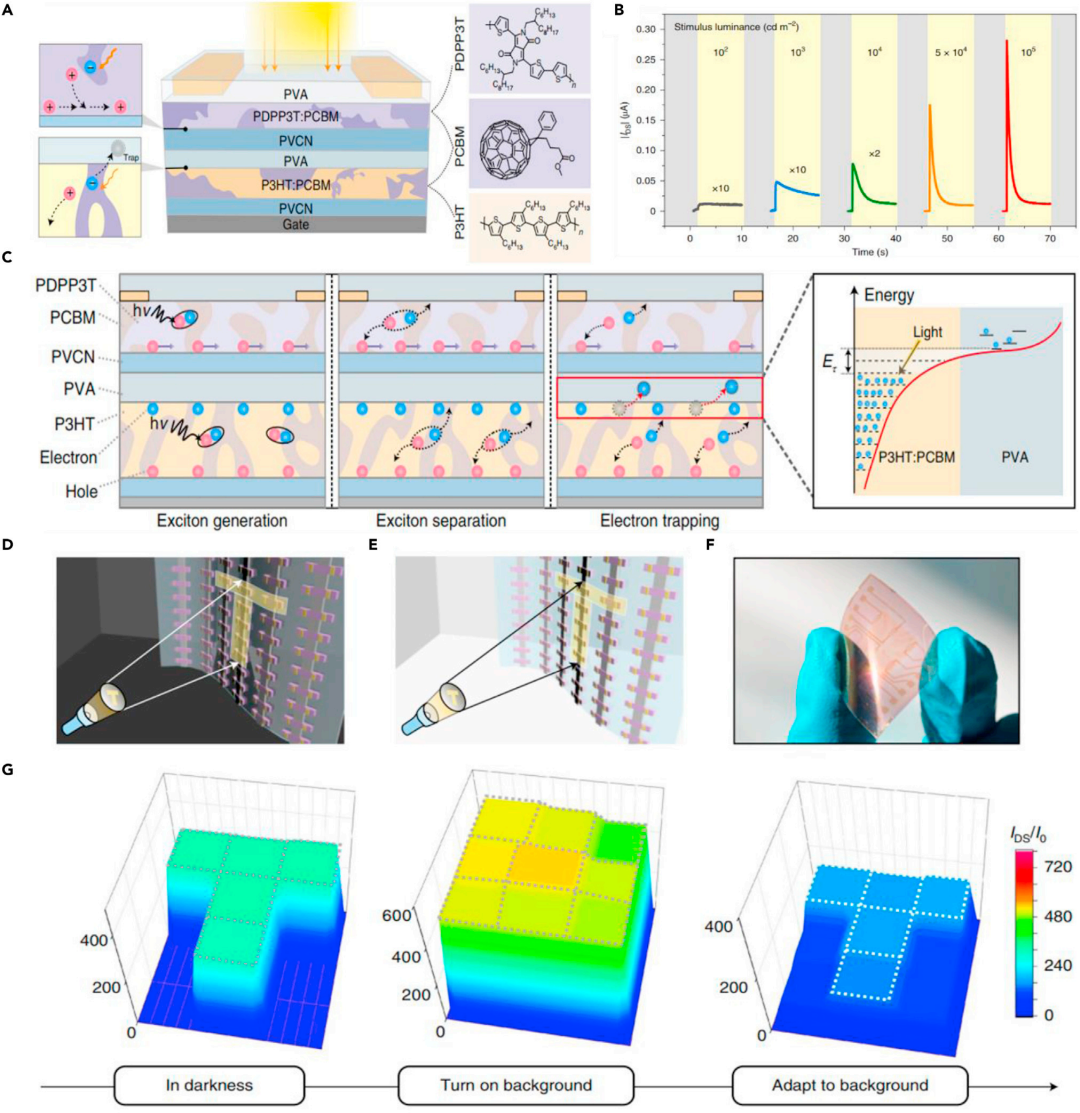
Organic material-based homogeneous integration machine vision system (A) Schematic illustration of the prepared BHJ organic transistor. (B) Real-time photoresponse of an organic transistor to various stimuli luminance. (C) Mechanism analysis of the optical response and adaptive process. (D and E) Conceptual designs of the organic transistor array in the dark and bright background. (F) Optical image of a flexible 3 × 3 organic transistor array. (G) Resulting current mapping of the organic transistor array under different conditions. Reproduced with permission (He et al., 2021). Copyright 2021, Springer Nature.

Excitingly, Zhu et al. reported a novel organic bulk heterojunction (BHJ) transistor array with excellent light intensity-dependent photoadaptation ability (He et al., 2021). The prepared devices simulate the transmembrane transport characteristics of Ca^2+^ and Na^+^ in the human cell membrane regulated by the feedforward mechanism. As shown in Figure 9A, there are two kinds of BHJ, *viz.,* poly{2,2′-[(2,5-bis(2-hexyldecyl)-3,6-dioxo-2,3,5,6-tetrahydropyrrolo[3,4-c]pyrrole-1,4-diyl) dithiophene]-5,5′-diyl-alt-thiophen-2,5-diyl} (PDPP3T):[6,6]-phenyl-C_61_-butyric acid methyl ester (PCBM) and Poly(3-hexylthiophene-2,5-diyl) (P3HT):PCBM, separated by two insulating layers, polyvinyl alcohol (PVA), and poly(vinyl-cinnamate) (PVCN). The upper BHJ is mainly used as the transport layer for photo-generated carriers and the lower serves as the floating gate to regulate the shielding effect of the bottom gate on the upper channel. It is worth mentioning that interface engineering is of great significance to affect the overall performance of the device. Compared with PVCN layer, there are a large number of defect states at the interface of PVA and lower BHJ, which could capture photo-generated electrons and shield the gate influence to a certain extent. As shown in Figure 9B, the prepared organic transistor exhibits excellent adaptability within a large range of light intensity. Moreover, mechanism analysis demonstrates the generation and separation of excitons and the transport and capture of carriers work together to create the adaptive characteristics of the device (Figure 9C). Furthermore, a 3 × 3 flexible organic transistor array was fabricated to verify the image processing capability in the real environment. As shown in Figures 9D–9G, a T-shaped optical pattern was projected onto the sensor array, which accounts for a T-shaped photocurrent response on the array. In addition, when the background is overexposed, the transistor array could shield the environment noise and highlight the real information within 2 s. Compared with the adaptation ability of the human eye, this array exhibits a faster adaptive speed, which provides a new idea for the construction of the artificial vision system. However, this device could only achieve in-situ filtering for strong light information but cannot realize the image enhancement function under dim conditions. Moreover, a higher-density and higher-resolution array is needed to improve the image quality and explore more abundant application scenarios.

## Challenges and outlook

By simulating the working principles of the human visual system, retinomorphic machine vision technology based on new materials, mechanisms, and architectures has achieved vigorous development in the artificial intelligence era (Cho et al., 2021; Wang et al., 2020b). In this contribution, we systematically summarized two typical retinomorphic optoelectronic device paradigms, *viz.,* heterogeneous and homogeneous integration architectures. Although much encouraging progress has been achieved, there remain challenges and opportunities in front of researchers.

At the material level, the development of large-scale, high-quality, and low-cost thin-film preparation technology is still an urgent problem to be solved, especially for 2D materials and perovskite materials (Lin et al., 2020; Liu et al., 2020; Zavabeti et al., 2020). Although several reported 2D materials have grown at the wafer scale, most of such attempts are still in the exploratory stage. Moreover, because the several reported 2D material transfer technologies are inefficient and the technical requirements for the operators are very high, it is imminent to prepare high-quality and large-size 2D materials directly on the selected substrate (Wang et al., 2021). In terms of improving the environmental stability of perovskites, appropriate protection strategies, *viz.,* physical encapsulation and chemical passivation are necessary (Gao et al., 2020; Lv et al., 2019). The former tends to protect the devices from eroding by water and oxygen, whereas the latter is dedicated to passivating the internal defects of the perovskites. However, the reported protection effects are still limited, more reliable and efficient approaches are needed to further expand their application prospects. For organic materials, the quality of organic films prepared with diverse process parameters is significantly different because of the inherent weak intermolecular interaction and disordered molecular entanglement manner, which accounts for cycle to cycle (C2C) and device-to-device (D2D) variations, such as threshold voltage, write/erase speed, number of states, state retention time, durability, etc (van de Burgt et al., 2018). Although several groups have devoted to improving the uniformity of the film through molecular planarization, the related research is still in its infancy (Zhang et al., 2021). Furthermore, different material systems have different compatibility with micro-nano processing technology, which deserves in-depth research for both academia and industry.

At the device level, more in-depth research about the physical mechanisms behind device performances and CMOS compatible integration strategies are necessary to purposefully improve overall performances before practical application. For image sensor units, optimizing device structure and energy level matching diagrams are effective approaches to improve the photoelectric conversion efficiency. Interface engineering also deserves more attention, which has a strong impact on the capture and transfer of carriers (Graetzel et al., 2012; Zhou et al., 2014). For memristor-based retinomorphic optoelectronic devices, the on-off of the conductive filament generally accounts for memristive switching. The movement of electrons or ions is random, which causes the inherent variability and instability of device conductance, especially in low conductivity regimes (Xia and Yang, 2019). Moreover, some researches only focus on whether the conductance of the synaptic device can be regulated with the pulse while ignoring the necessary nonvolatility of each conductivity state, which is indeed a key element for in-memory computing. Under the premise of accurately characterizing the memristive mechanism of the device, developing novel strategies to precisely control the internal ion transport engineering to improve the uniformity and reliability of the devices is still the main research direction in the future. Furthermore, solving the cross-talk problem of the memristor crossbar array is another difficulty. The existing transistor and selector technologies cannot be perfectly compatible with the memristor system without loss of performance, thus developing more refined peripheral control circuits is of great significance (Shi et al., 2020). It is worth mentioning that different material systems have different compatibility with the current CMOS-based micro-nano processing technology, which deserves in-depth research for both academia and industry.

In terms of two different integrated architectures, homogeneous integration has broader application prospects in the future. Although the functional separation mode of the heterogeneous architecture is beneficial to improving the performance of the independent unit, the infamous von Neumann's bottleneck still remains. High-throughput data transfer between the image sensor unit and front-end information storage and preprocessing module is bound to weaken the comprehensive performance of the device. However, homogeneous integration also faces huge challenges. More efficient device integration structures (not limited to crossbar arrays) and more complete external circuit servo systems will give a full play to its functions.

For applications, intelligent machine vision technology requires more consideration of complex and changeable environmental factors, such as different light intensities, the similarity between the background and the observed entity, and fast capture of moving objects, etc. Many influencing factors are difficult to be simulated in the laboratory and can only be verified through practical applications. Therefore, a more stringent device performance evaluation system is needed to guide the development of the industry. In addition, although the current artificial neural networks could solve simple image processing problems, their efficiency decreases while energy consumption increases when facing complex tasks. To further simulate the way that the human brain works, more efficient neural network architectures are necessary.
